# β-catenin attenuation leads to up-regulation of activating NKG2D ligands and tumor regression in *Braf^V600E^*-driven thyroid cancer cells

**DOI:** 10.3389/fimmu.2023.1171816

**Published:** 2023-07-06

**Authors:** Minjing Zou, Suhad Al-Yahya, Monther Al-Alwan, Huda A. BinEssa, Khalid S. A. Khabar, Falah Almohanna, Abdullah M. Assiri, Abdulmohsen Altaweel, Amal Qattan, Brian F. Meyer, Ali S. Alzahrani, Yufei Shi

**Affiliations:** ^1^ Department of Molecular Oncology, King Faisal Specialist Hospital and Research Centre, Riyadh, Saudi Arabia; ^2^ Department of Molecular Biomedicine, King Faisal Specialist Hospital and Research Centre, Riyadh, Saudi Arabia; ^3^ Department of Stem Cell & Tissue Re-engineering, King Faisal Specialist Hospital and Research Centre, Riyadh, Saudi Arabia and College of Medicine, Al-Faisal University, Riyadh, Saudi Arabia; ^4^ Department of Comparative Medicine, King Faisal Specialist Hospital and Research Centre, Riyadh, Saudi Arabia; ^5^ Mawhiba, King Abdulaziz and His Companions Foundation for Giftedness and Creativity, Riyadh, Saudi Arabia; ^6^ Centre for Genomic Medicine, King Faisal Specialist Hospital and Research Centre, Riyadh, Saudi Arabia; ^7^ Department of Medicine, King Faisal Specialist Hospital and Research Centre, Riyadh, Saudi Arabia

**Keywords:** β-catenin, *CTNNB1*, *BRAF^V600E^*, NKG2D ligands, NK cells, thyroid cancer

## Abstract

**Introduction:**

*BRAF^V600E^* mutations frequently occur in papillary thyroid cancer (PTC). β-catenin, encoded by *CTNNB1*, is a key downstream component of the canonical Wnt signaling pathway and is often overexpressed in PTC. *BRAF^V600E^*-driven PTC tumors rely on Wnt/β-catenin signaling to sustain growth and progression.

**Methods:**

In the present study, we investigated the tumorigenicity of thyroid cancer cells derived from *BRAF^V600E^* PTC mice following *Ctnnb1* ablation (BVE-*Ctnnb1^null^*).

**Results:**

Remarkably, the tumorigenic potential of BVE-*Ctnnb1^null^* tumor cells was lost in nude mice. Global gene expression analysis of BVE-*Ctnnb1^null^* tumor cells showed up-regulation of NKG2D receptor activating ligands (H60a, H60b, H60c, Raet1a, Raet1b, Raet1c, Raet1d, Raet1e, and Ulbp1) and down-regulation of inhibitory MHC class I molecules H-2L and H-2K2 in BVE-*Ctnnb1^null^* tumor cells. *In vitro* cytotoxicity assay demonstrated that BVE-*Ctnnb1^wt^* tumor cells were resistant to NK cell-mediated cytotoxicity, whereas BVE-*Ctnnb1^null^* tumor cells were sensitive to NK cell-mediated killing. Furthermore, the overexpression of any one of these NKG2D ligands in the BVE-*Ctnnb1^wt^* cell line resulted in a significant reduction of tumor growth in nude mice.

**Conclusions:**

Our results indicate that active β-catenin signaling inhibits NK cell-mediated immune responses against thyroid cancer cells. Targeting the β-catenin signaling pathway may have significant therapeutic benefits for *BRAF*-mutant thyroid cancer by not only inhibiting tumor growth but also enhancing host immune surveillance.

## Introduction

Papillary thyroid cancer (PTC) is the most common type of thyroid cancer, accounting for more than 80% of all thyroid cancer cases (1). The *BRAF^V600E^* mutation is the most frequent genetic alteration in PTC ranging from 29% to 83% depending on the cohort studied with overall rate of approximately 45% (2, 3). Nearly 60% (59.7% of 496 cases) of PTC patients harbor *BRAF^V600E^* mutations from the TCGA thyroid cancer consortium (4). In some studies, *BRAF* ^V600E^ mutation has been shown to be one of the factors contributing to thyroid cancer recurrence and mortality (5, 6). The mutation constitutively activates the RAS–RAF–MEK–ERK mitogen-activated protein kinase (MAPK) signaling cascade and promotes the initiation and progression of PTC. It has been demonstrated that *Braf* ^V600E^ mutation drives oncogenic transformation of thyroid epithelial cells and development of PTC in transgenic mouse models (7, 8).

β-catenin is a downstream component of the Wnt/β-catenin pathway and plays an important role in embryonic development and tissue homeostasis (9). Dysregulation of its signaling is involved in many types of tumors, including thyroid cancer (10–13). Functional cross-talk between β-catenin and MAPK, PI3K/AKT, or CREB (cAMP-response element binding protein) signaling pathways has been demonstrated to maintain proliferation in thyroid cancer cell lines (12, 14). Recently, we have shown that *BRAF*-driven PTC requires active β-catenin signaling to sustain its growth (15). Deletion of β-catenin results in tumor regression and differentiation as well as increased sensitivity to the BRAF^V600E^ inhibitor PLX4720.

In this study, we investigated the tumorigenicity of thyroid cancer cells derived from *Braf^V600E^* -induced PTC mice with wild-type *Ctnnb1* (BVE-*Ctnnb1* ^wt^) and deleted *Ctnnb1* (BVE-*Ctnnb1* ^null^). We found that β-catenin ablation led to complete loss of tumorigenicity of BVE-*Ctnnb1* ^null^ cells in nude mice, with increased expression of NKG2D ligands in BVE-*Ctnnb1* ^null^ cells. Our data strongly suggest that β-catenin exerts oncogenic effects by promoting tumor growth and evading NK cell-mediated immune surveillance.

## Materials and methods

### Animals

Athymic BALB/c-nu/nu (nude mice) and C57BL/6J mice (6–10 weeks of age) were acquired from Jackson Laboratory. Mice were provided with autoclaved food and water ad libitum. The study was approved by the Animal Care and Use Committee of the institution (RAC# 2190004) and was conducted in compliance with the Public Health Service Guidelines for the Care and Use of Animals in Research.

### Thyroid tumor cell lines

BVE*-Ctnnb1* ^wt^ and BVE-*Ctnnb1* ^null^ were established from thyroid tumors collected aseptically from donor mice, as described previously (15) and maintained in DMEM/F12 growth medium containing 10% fetal bovine serum, 100 units/ml penicillin, and 100 μg/ml streptomycin.

### RNA sequencing for quantification of differentially expressed genes

Total RNA from BVE*-Ctnnb1* ^wt^ and BVE-*Ctnnb1* ^null^ cell lines was isolated using TRI Reagent® solution (#T9424, Sigma-Aldrich, St. Louis, MO, USA). Libraries were constructed using an Illumina TruSeq RNA Library Prep kit (San Diego, CA, USA) according to the manufacturer’s instructions. All sequencing was performed on an Illumina HiSeq 4000 platform with at least 20 million clean reads. Significant DEGs were selected based on the following criteria: Log2 fold change>2, false discovery rate (FDR) <0.001, and p-value from the difference test<0.01. Gene list annotation and enrichment of biological pathways were performed using Metascape (16).

### Cytotoxicity assay and phenotyping of effector cells

A label-free cell-based assay (ACEA Biosystems, San Diego, CA, USA) was used to measure the cytotoxicity of spleen cells from C57BL/6J (effector) mice against tumor cells (target) at an effector/target ratio of 25:1, as previously described (17). The effector cells were immune phenotyped for the presence of surface markers: CD4, CD8, and asialo GM1 (aGM1) using complement-mediated (Cederlane Low-Tox®-M Rabbit Complement, #CL3051, 1:20 dilution) cytotoxicity assay in the presence or absence of appropriate blocking antibodies (18). Briefly, target cells (2000 cells/well) were seeded in electrode strip-coated 96-well electrode-integrated microplates. Spleen cells were harvested from two normal C57BL/6J mice. Splenocytes were added to the target cells at 50,000 cells/well in the presence of 5 µg/ml phytohemagglutinin (PHA) and 100 U/ml IL-2 for the activation of T and NK cells with or without anti-CD8 (#CL169AP, 1:20 dilution, plus 5ul complement), anti-CD4 (#CL012AP, 1:20 dilution, plus 5ul complement), or asialo GM1 (anti-NK cell) antibodies (#CL8955, 1:100 dilution, plus 5ul complement, Cedarlane, Ontario, Canada). The percentage of specific cytotoxicity was calculated based on the cell index (CI), which was derived from the changes in electrode impedance due to cell growth and attachment relative to that of the background (without cells). The following formula was used: Cytotoxicity = [(experimental CI – control CI)/(untreated CI – control CI)] × 100.

### Cloning and expression of activating NKG2D ligands in BVE-*Ctnnb1* ^wt^ cells

The cDNA for each NKG2D ligand H60a (NM_010400.2), H60b (NM_001177775.1), H60c (ENSMUST00000170893.2), Rae-1α (Raet1a, NM_009016.1), Rae-1β (Raet1b, NM_009017.1), Rae-1γ (Raet1c, NM_009018.1), Rae-1δ (Raet1d, NM_020030.2), Rae-1ϵ (Raet1e, NM_001359808.1) or Ulbp1 (Mult1, NM_029975.2) was cloned into pcDNA3.1 expression vector and transfected into the BVE-*Ctnnb1* ^wt^ cell line using Lipofectamine (Invitrogen, CA, USA). The transfected cells were selected for 6 weeks with 100 µg/ml zeocin. Stable clones expressing each ligand were subcutaneously injected into nude mice to observe tumor growth. The overexpression of NKG2D ligands was confirmed by qRT-PCR.

### Cloning and expression of inhibitory Ly49 ligands in BVE-*Ctnnb1* ^null^ cells

The cDNA for each Ly49 ligand H2-L, H2-K2, and H2-K1 was cloned into the pcDNA3.1 expression vector and transfected into the BVE-*Ctnnb1* ^null^ cell line using Lipofectamine (Invitrogen, CA, USA) and selected for 6 weeks with 100 µg/ml zeocin. Stable clones expressing each ligand were subcutaneously injected into nude mice to observe tumor growth. The overexpression of H2-L, H2-K2, and H2-K1 was confirmed by qRT-PCR.

### Statistical analysis

Student’s *t*-test (two-tailed) was used to compare two groups and the area under curve (AUC) calculation was performed using GraphPad Prisim 8. A *P* value < 0.05 was considered significant.

## Results

### Tumorigenicity of BVE-*Ctnnb1* ^null^ cells

**Figure 1:**
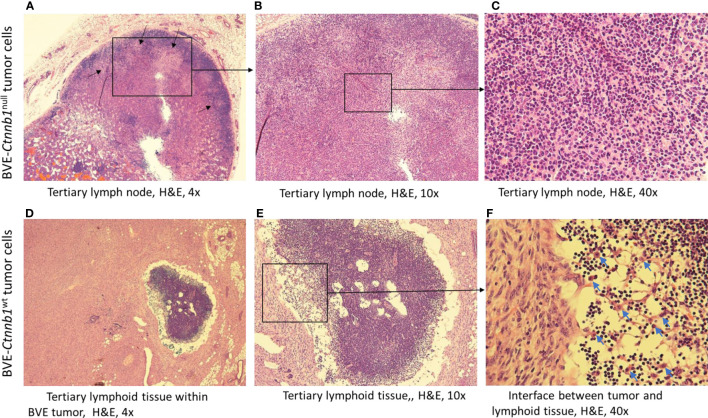
Tertiary lymph node formation at the injection site following subcutaneous injection of BVE-*Ctnnb1* ^null^ tumor cells into nude mice. Upper **(A–C)** (H&E staining): Characteristic features of lymphoid follicles and germinal centers are shown. Germinal centers are indicated by an arrow; Lower **(D–F)**: Tertiary lymphoid tissue within a large tumor following subcutaneous injection of BVE-*Ctnnb1* ^wt^ tumor cells into nude mice. Heavy infiltration of lymphocytes and macrophages at the interface between tumor and lymphoid tissue are shown.

Tumorigenicity of BVE-*Ctnnb1* ^null^ and BVE-*Ctnnb1* ^wt^ cells was investigated in athymic nude mice. No tumor growth was observed after 4 weeks of subcutaneous injection of 1 × 10^6^ BVE-*Ctnnb1* ^null^ cells into the right flank of the mice (n=8), except that a small nodule (0.5 × 0.5 cm) was formed at the injection site in one mouse (n=8). However, tumors (approximately 2 × 2 cm) developed in all mice 4 weeks following injection of 1 × 10^6^ BVE-*Ctnnb1* ^wt^ cells. The nodule, which formed at the injection site of BVE-*Ctnnb1* ^null^ cells, was confirmed to be a tertiary lymph node by histology, with characteristic features of lymphoid follicles and germinal centers (**Figures 1A-C**; **Supplementary Figure 1**). Lymphoid tissues were also found within the tumors formed by BVE-*Ctnnb1* ^wt^ tumor cells, with heavy infiltration of lymphocytes and macrophages at the interface of the tumor and lymphoid tissue (**Figures 1D-F**; **Supplementary Figure 1**).

### Overexpression of NKG2D receptor activating ligands in the BVE-*Ctnnb1* ^null^ cells

**Figure 2:**
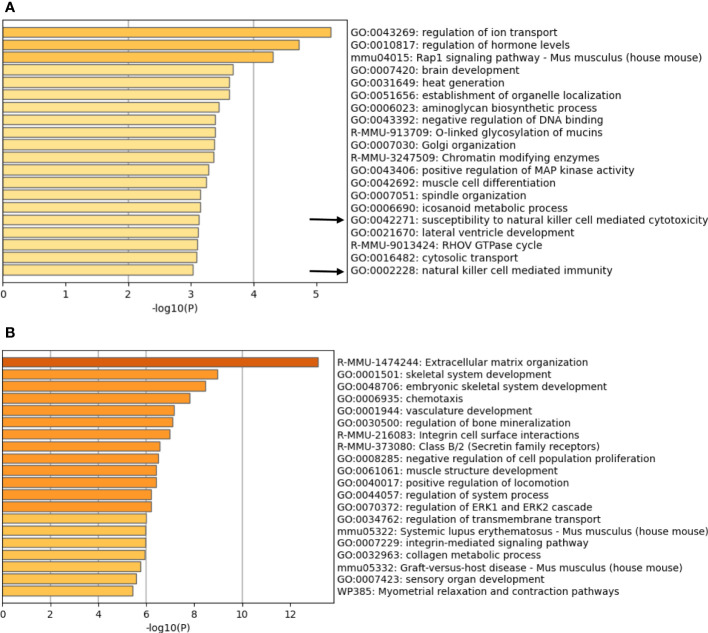
Enriched ontology clusters of differentially expressed genes. Pathway analysis of **(A)** up-regulated genes (833 genes) and **(B)** down-regulated genes (1407 genes) in the BVE-*Ctnnb1* ^null^ cells as compared to BVE-*Ctnnb1* ^wt^ cells. Top 20 enriched terms (GO/KEGG terms, canonical pathways, hall mark gene sets) are shown and colored by p-values. The gene clusters involving NK cell mediated immunity are indicated by an arrow.

Since nude mice have no functional T cells, the rejection of BVE-*Ctnnb1* ^null^ cells is likely mediated by NK cells. To explore the mechanisms that resulted in BVE-*Ctnnb1* ^null^ tumor cell rejection, we compared the gene expression profiles of BVE-*Ctnnb1* ^wt^ and BVE-*Ctnnb1* ^null^ cells using RNA-Seq analysis of three biological replicates. Pathway analysis showed 833 up-regulated and 1407 down-regulated genes with a log2 fold-change >2 (**Supplementary Table 1**). Importantly, the genes involved in NK cell-mediated immunity were among the top 20 enriched ontology clusters that were significantly up-regulated in the BVE-Ctnnb1^null^ cells (**Figure 2A**). The genes typically involved in cancer growth and progression, such as extracellular matrix organization, chemotaxis, vascular development, integrin cell surface interactions and signaling, and ERK-1/ERK2 signaling, were among the top 20 enriched ontology clusters that were significantly down-regulated in the BVE-*Ctnnb1* ^null^ cells (**Figure 2B**). These changes in biological pathways are consistent with our previous study showing that the β-catenin signaling pathway is required for the growth and progression of BRAF^V600E^-driven thyroid cancer (15). The gene list for each cluster is presented in **Supplementary Tables 2** and **3**.

**Figure 3:**
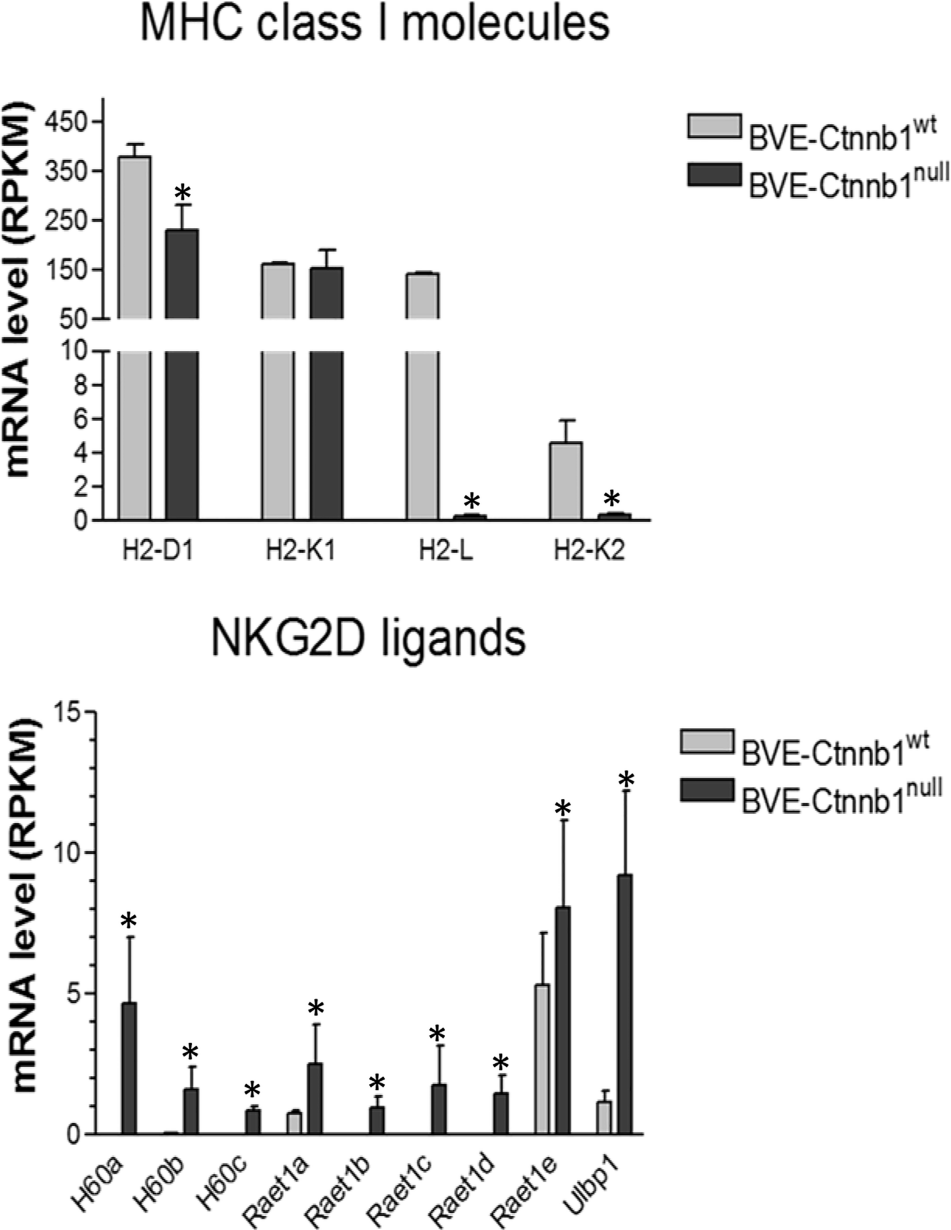
Expression of MHC class 1 molecules and NKG2D ligands in BVE-*Ctnnb1* ^wt^ and BVE-*Ctnnb1* ^null^ tumor cells. RNA-Seq analysis was performed on tumor cells and data are presented as mean ± SEM of three biological replicates. Down-regulation of MHC class 1 molecules (H2-L, H2-K2, and H2-D1), and up-regulation of NKG2D ligands. Asterisk indicates statistical significance as compared to BVE-Ctnnb1^wt^ control. RPKM indicates Reads Per Kilobase Million.

We further analyzed the list of DEGs involved in NK cell activation. As shown in **Figure 3**, BVE-*Ctnnb1* ^null^ cells showed up-regulation of NKG2D activating ligands (*H60a*, *H60b*, *H60c*, *Raet1a*, *Raet1b*, *Raet1c*, *Raet1d*, *Raet1e* and *Ulbp1*) and down-regulation of inhibitory MHC class I molecules (H2-L, H2-K2, and H2-D1), which may lead to NK cell activation and tumor cell clearance. In contrast, the up-regulation of H2-L and H2-K2 and down-regulation of NKG2D ligands in BVE-*Ctnnb1* ^wt^ tumor cells would probably result in evasion of NK cell-mediated immune surveillance and promotion of tumor growth.

### NK-mediated cytotoxicity of BVE-*Ctnnb1* ^wt^ and BVE-*Ctnnb1* ^null^ cells

**Figure 4:**
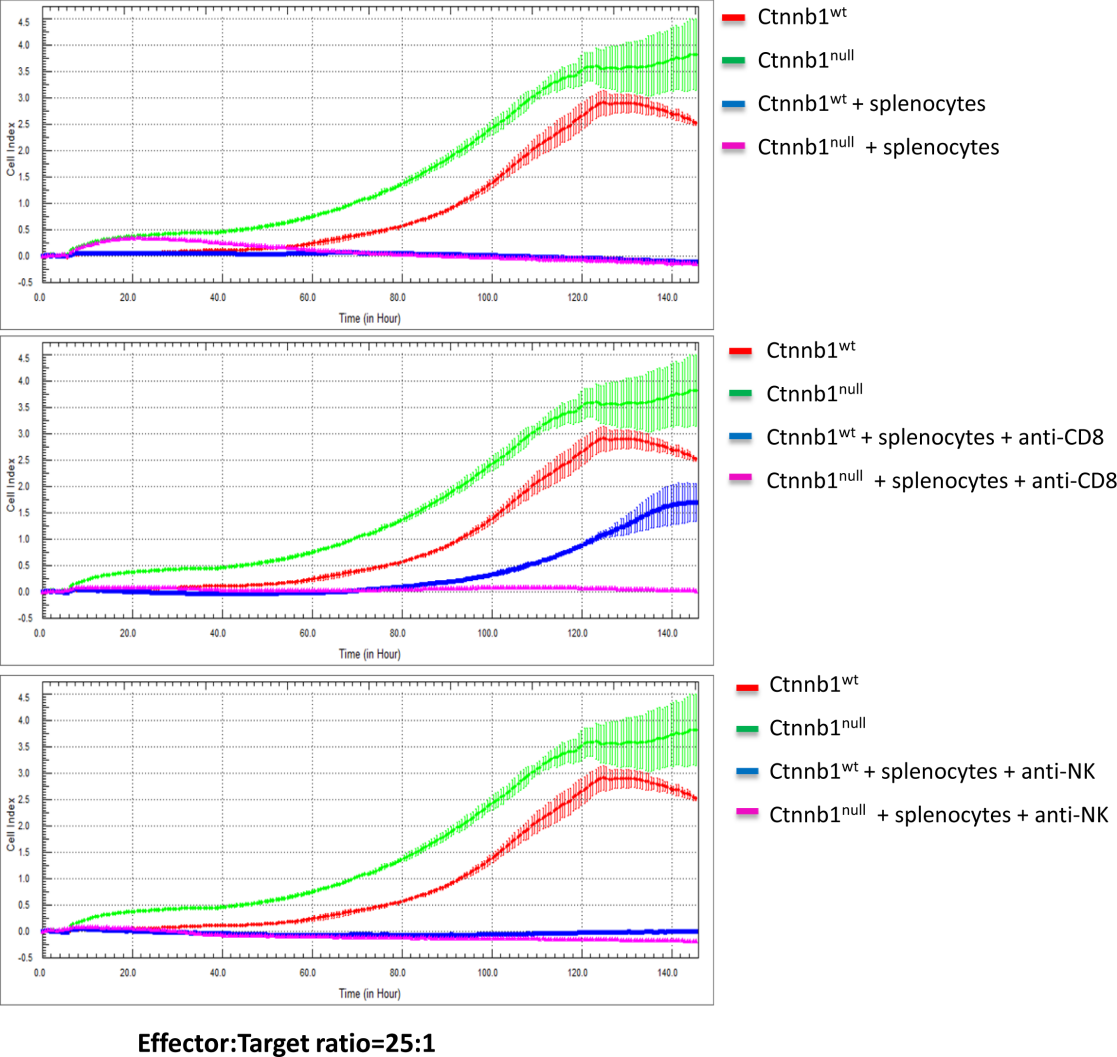
Cytotoxicity of splenocytes against BVE-*Ctnnb1* ^wt^ and BVE-*Ctnnb1* ^null^ tumor cells. The cytotoxicity of spleen cells (effector) against tumor cells (target) was measured at an effector/target ratio of 25:1 by real-time *in vitro* cytolytic assay in the presence or absence of anti-CD8 or anti-NK cell antibodies. The data are presented as the Cell Index over time. The target cell proliferation rate was registered as an increase in the impedance-related Cell Index over time. When effectors are added to target cells, their cytolytic activity causes adherent target cells to round up and detach, consequently reducing the Cell Index value.

Next, we investigated the sensitivity of BVE-*Ctnnb1* ^null^ and BVE-*Ctnnb1* ^wt^ cells to NK cell-mediated cytotoxicity using *in vitro* cytotoxicity assay. As shown in **Figure 4**, activated splenocytes completely inhibited the replication of both BVE-*Ctnnb1* ^wt^ and BVE-*Ctnnb1* ^null^ tumor cells (top panel). However, BVE-*Ctnnb1* ^wt^ tumor cells were able to replicate at 67% efficiency at 120h in the presence of anti-CD8 T cell neutralizing antibodies, indicating their resistance to NK cell-mediated cytotoxicity. The area under curve (AUC) of BVE-*Ctnnb1* ^null^, BVE-*Ctnnb1* ^wt^, and BVE-*Ctnnb1* ^wt^ + splenocytes + anti-CD8 were 210.4 ± 3.2, 128.7 ± 2.4, and 42.5 ± 1.9, respectively. In contrast, BVE-*Ctnnb1* ^null^ tumor cells were unable to grow in the presence of anti-CD8 T cell neutralizing antibodies, indicating their sensitivity to NK killing (middle panel). Both BVE-*Ctnnb1* ^wt^ and BVE-*Ctnnb1* ^null^ tumor cells were unable to replicate in the presence of anti-NK cell neutralizing antibodies, indicating their sensitivity to CD8 T cell-mediated cytotoxicity (bottom panel).

### Overexpression of NKG2D ligands in BVE-*Ctnnb1* ^wt^ cells reduces tumor growth

**Figure 5:**
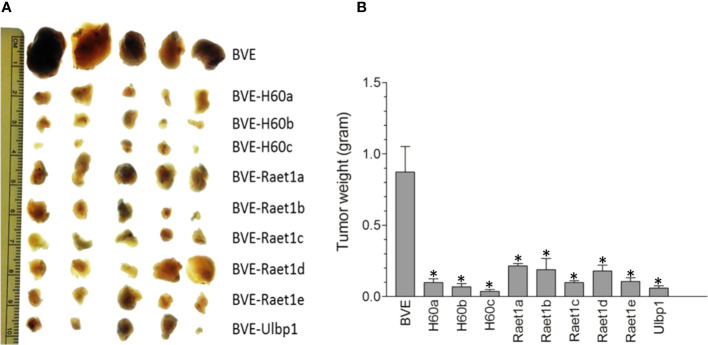
Regression of tumors after overexpression of NKG2D ligands in the BVE-*Ctnnb1* ^wt^ cells. **(A)** Each of NKG2D ligands was stably transfected under CMV promoter into BVE-*Ctnnb1* ^wt^ cells for stable expression. These NKG2D ligand-expressing cells (1 x 10^6^) were injected subcutaneously into nude mice (n=5) to observe tumor growth for 4 weeks. **(B)** Quantification of tumor growth (weight) after subcutaneous injection of each NKG2D ligand-expressing BVE-*Ctnnb1* ^wt^ cells. Data are expressed as mean of tumor weight ± SEM of 5 tumors. Asterisk indicates statistical significance as compared to BVE-Ctnnb1^wt^ control.

To further confirm the role of NKG2D activating ligands in tumor regression, we overexpressed each of these ligands by stable transfection of their cDNAs into BVE-*Ctnnb1* ^wt^ cells and then injected these ligand-expressing tumor cells (1 × 10^6^) into nude mice (n=5) to observe tumor growth for 4 weeks. As shown in **Figure 5**, overexpression of these ligands caused a significant reduction in tumor growth (p<0.001). In contrast to the complete loss of tumorigenicity in BVE-*Ctnnb1* ^null^ cells, over-expression of a single NKG2D ligand could not eliminate BVE-*Ctnnb1* ^wt^ tumor cell growth in nude mice. This might be due to high expression of inhibitory H2-L and H2-K2 ligands in BVE-*Ctnnb1* ^wt^ tumor cells and/or tumor cell clearance may require the simultaneous expression of multiple NKG2D ligands for optimal NK cell activation.

### Overexpression of Ly49 ligands in BVE-*Ctnnb1* ^null^ cells partially rescue tumor growth

**Figure 6:**
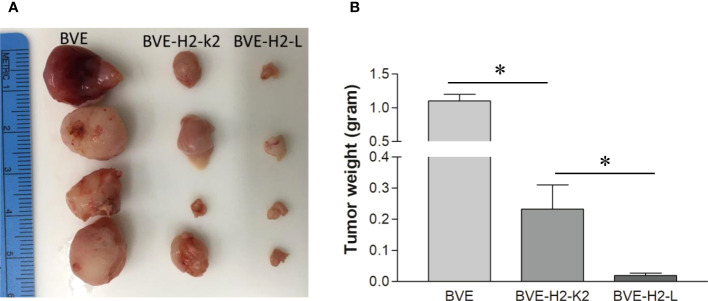
Tumor formation after overexpression of MHC class 1 molecules H2-L and H2-K2 in BVE-*Ctnnb1* ^null^ tumor cells. **(A)** Each of MHC class 1 molecules H2-L and H2-K2 was stably transfected under CMV promoter into BVE-*Ctnnb1* ^null^ cells. These H2-L and H2-K2-expressing cells (1 x 10^6^) were injected subcutaneously into nude mice (n=4) to observe tumor growth for 6 weeks. BVE (BVE-*Ctnnb1* ^wt^) cells were used as control. **(B)** Quantification of tumor weight after subcutaneous injection of H2-L and H2-K2-expressing BVE-*Ctnnb1* ^null^ cells to nude mice. Data are expressed as mean of tumor weight ± SEM of 4 tumors. Asterisk indicates statistical significance as compared to BVE-Ctnnb1^wt^ control or between BVE-H2-K2 and BVE-H2-L.

**Figure 7:**
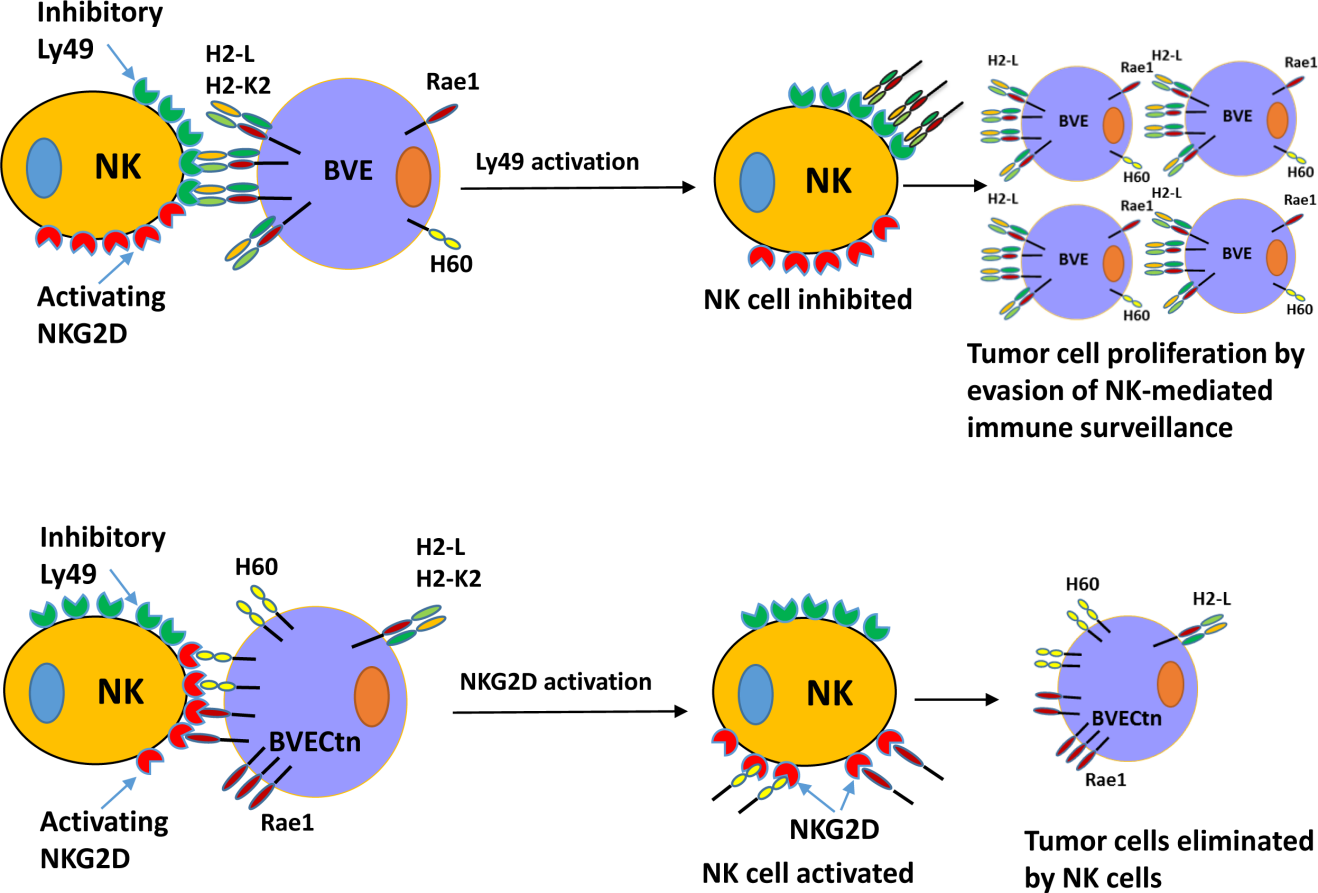
Schematic diagram of NK cell-mediated tumor rejection in nude mice. Top: BVE (BVE-*Ctnnb1* ^wt^) tumor cells express high levels of MHC class I molecules H2-L and/or H2-K2, which bind to the inhibitory Ly49 receptor on the NK cells. This interaction results in NK cell inhibition and impaired immune surveillance. Bottom: BVECtn (BVE-*Ctnnb1* ^null^) tumor cells express high levels of NKG2D receptor ligands H60 (a, b, and c) and Raet1 (a, b, c, d, and e), which bind to the activating NKG2D receptor. This binding leads to NK cell activation and tumor cell rejection.

Since BVE-*Ctnnb1* ^null^ cells express high levels of NKG2D activating ligands and low levels of H2-L and H2-K2 inhibitory ligands, we investigated whether the loss of tumorigenicity in BVE-*Ctnnb1* ^null^ cells could be partially rescued by overexpression of H2-L or H2-K2 ligands in these cells. As shown in **Figure 6**, overexpression of these ligands resulted in tumor growth in nude mice, thus confirming the role of H2-L and H2-K2 ligands in NK cell inhibition. H2-K2 appeared to be more effective than H2-L in inhibiting NK cells. The potential mechanisms of NK cell-mediated rejection of BVE-*Ctnnb1* ^null^ cells are summarized in **Figure 7**.

## Discussion

In the present study, we provided evidence of β-catenin in modulating NK cell activity against *Braf*-mutant PTC in mice. Down-regulation of β-catenin increased the expression of NKG2D ligands and decreased the expression of inhibitory MHC class I molecules, such as H-2L and H-2K2, in tumor cells, which triggered NK cell-mediated tumor rejection.

It has been reported that oncogenic activation of KRAS/BRAF/MEK signaling stimulates Wnt/β-catenin pathway by β-catenin/TCF4 complex activation via Frizzled co-receptor LRP6 phosphorylation, which in turn promotes tumor growth and invasion (19, 20). The LRP6 may be the link between MAPK and Wnt/β-catenin signaling cross-talk during oncogenesis (20). In the BrafV600E murine thyroid cancer, β-catenin expression is up-regulated and its knockout results in down-regulation of MARK signaling pathway, indicating a positive crosstalk between MAPK and Wnt/β-catenin signaling pathways. Active Wnt/β-catenin signaling in turn promotes *Braf^V600E^*-mediated tumor growth (15). Genetic alterations in β-catenin have not been identified in PTC. However, aberrant β-catenin expression or localization, such as the reduction of β-catenin plasma membrane levels and its aberrant nuclear localization, has been reported and is associated with c-Myc and cyclin D1 overexpression, loss of tumor differentiation, and poor prognosis in human PTC samples (13, 21).

The activation of tumor-intrinsic Wnt/β-catenin signaling is frequently associated with poor prognosis in most human cancers. Besides its intrinsic effects on tumor cell proliferation and apoptosis, the WntT/β-catenin signaling pathway has been found to act on the tumor microenvironment and lead to immune evasion by reducing tumor infiltration of dendritic cells (DCs), NK cells, and T cells via multiple mechanisms, such as inhibition of CCL4 expression on cytotoxic T lymphocytes, CXCL10 expression on DCs, or increased expression of dickkopf WNT signaling pathway inhibitor 1 (DKK1), which prevents NK cell-dependent cancer cell lysis by reducing the expression of NK cell-activating ligands (22–24).

Lee et al. reported that incubation of NK cells and lymphokine-activated killer cells with TGF-β1 resulted in a dramatic reduction in surface NKG2D expression associated with impaired NK cytotoxicity (25). They further demonstrated that the modulation of NKG2D by TGF-β1 was specific, as the expression of other NK receptors was not affected by the presence of TGF-β1. TGF-β1-mediated impairment of NK cell cytotoxicity was not due to alteration of other lytic moieties, such as perforin, Fas, or the apoptotic pathway. TGF-β1 signaling is activated in BRAFV600E-induced thyroid cancer (26). In our previous studies, we showed that TGF-β inhibited the anti-tumor activity of NK and CD8+ T cells in *Braf^V600E^* thyroid cancer mouse model. The immune suppression could be reversed by IL-12 treatment with increased lymphocyte and macrophage infiltration, resulting in a significant reduction in tumor load and an increase in survival (18). Increased infiltration of lymphocytes and macrophages has also been observed in the same thyroid cancer model following β-catenin ablation (15). Spranger et al. have reported that active β-catenin signaling in melanoma prevents anti-tumor immunity via depletion of T cells, linking β-catenin signaling to tumor evasion of immune surveillance (23, 27). Wang et al. have recently shown that β-catenin inhibition shifts the colorectal tumor microenvironment into a T cell-inflamed phenotype through the up-regulation of T/NK cell-recruiting CXCR3 chemokines CXCL9/10/11 (28).

In the present study, we have identified a novel mechanism by which *Braf^V600E^*-mutant thyroid cancer cells evade NK cell-mediated immune surveillance. This is achieved by down-regulation of stimulating NKG2D ligands and up-regulation of inhibitory MHC class I molecules. Given that the activation of Wnt/β-catenin signaling leads to increased TGF-β signaling, the down-regulation of stimulating NKG2D ligands and up-regulation of inhibitory MHC class I molecules may be due to increased TGF-β signaling. However, in contrast to the reduction in surface NKG2D expression reported by Lee et al, NKG2D expression was not significantly different between BVE-*Ctnnb1* ^wt^ and BVE-*Ctnnb1* ^null^ tumor cells.

NK cells play an important role in cancer immune surveillance because of their ability to recognize tumor cells through the interaction of several distinct cell surface receptors, with the net effect of NK cell activation and cytotoxic attack on tumor cells (29, 30). The activation of NK cells **(**CD56+ in humans and CD49b+ in mice) is balanced by anti- and pro-activation signals through inhibitory and activating receptors, such as Ly49 in mouse or killer cell Ig-like receptor (KIR in human) family receptors, NKG2 (CD159) family of C-type lectin-like receptors, including activating NKG2D and inhibitory NKG2A, and natural cytotoxicity receptors (NKp46, NKp44, and NKp30) (31–33). NKG2D is expressed in all NK cells and activated CD8+ T cells. Its ligands are not or rarely expressed in normal tissues, but are expressed in tumor cells and viral-infected tissues (34). Previous studies have shown that the expression of NKG2D ligands on tumor cells renders them susceptible to killing by NK cells *in vitro* and the rejection of tumor xenografts *in vivo* (35, 36). NKG2D-mediated killing is negatively regulated by MHC class I molecules on tumor cells, which bind to inhibitory Ly49 receptors on NK cells to maintain a balance between activating and inhibitory signals. Thus, high expression of MHC class I molecules in tumor cells prevents efficient NKG2D-mediated cytotoxicity against tumor cells (37, 38). Our data indicate that active β-catenin signaling in *Braf^V600E^*-mutant thyroid cancer cells inhibits NK cell-mediated immune response. Attenuation of Wnt/β-catenin signaling boosts the host immune response against thyroid cancer. To our knowledge, this is a first report describing the inhibitory effect of β-catenin expression on NK cell activity, linking oncogene activation to innate immune suppression. Given that overexpression of NKG2D ligands results in regression of thyroid cancer xenografts *in vivo*, these ligands could be used for cancer vaccine development or gene therapy to generate anti-cancer immune responses.

In summary, our results provide insights into β-catenin signaling in the NK cell-mediated immune response against *BRAF*-mutant thyroid cancer cells. β-catenin ablation results in increased expression of activating NKG2D ligands and decreased expression of inhibitory MHC class I molecules, H2-L and H2-K2. Targeting the β-catenin signaling pathway could not only inhibit tumor growth but also enhance host immune surveillance.

## Data availability statement

The datasets presented in this study can be found in online repositories. The names of the repository/repositories and accession number(s) can be found below: https://www.ncbi.nlm.nih.gov/geo/query/acc.cgi?acc=GSE207695.

## Ethics statement

The animal study was reviewed and approved by The Animal Care and Use Committee of King Faisal Specialist Hospital and Research Centre.

## Author contributions

MZ, ASA, MA-A and YS contributed to conception and design of the study. MZ, SA-Y, MA-A, HB, KK, FA, and AMA performed experiments. AQ organized the database. MZ and YS performed the statistical analysis. MZ wrote the first draft of the manuscript. MA-A, BM, ASA, and YS wrote sections of the manuscript. All authors contributed to manuscript revision, read, and approved the submitted version.
