# Supplementary material for: β-catenin attenuation leads to up-regulation of activating NKG2D ligands and tumor regression in BrafV600E -driven thyroid cancer cells
Source: Front Immunol. 2023 Jul 6;14:1171816. doi: 10.3389/fimmu.2023.1171816 (PMC10358762; doi:10.3389/fimmu.2023.1171816)
Supplement: Supplementary file 1 [file Presentation_1.pptx]

## Slide 1
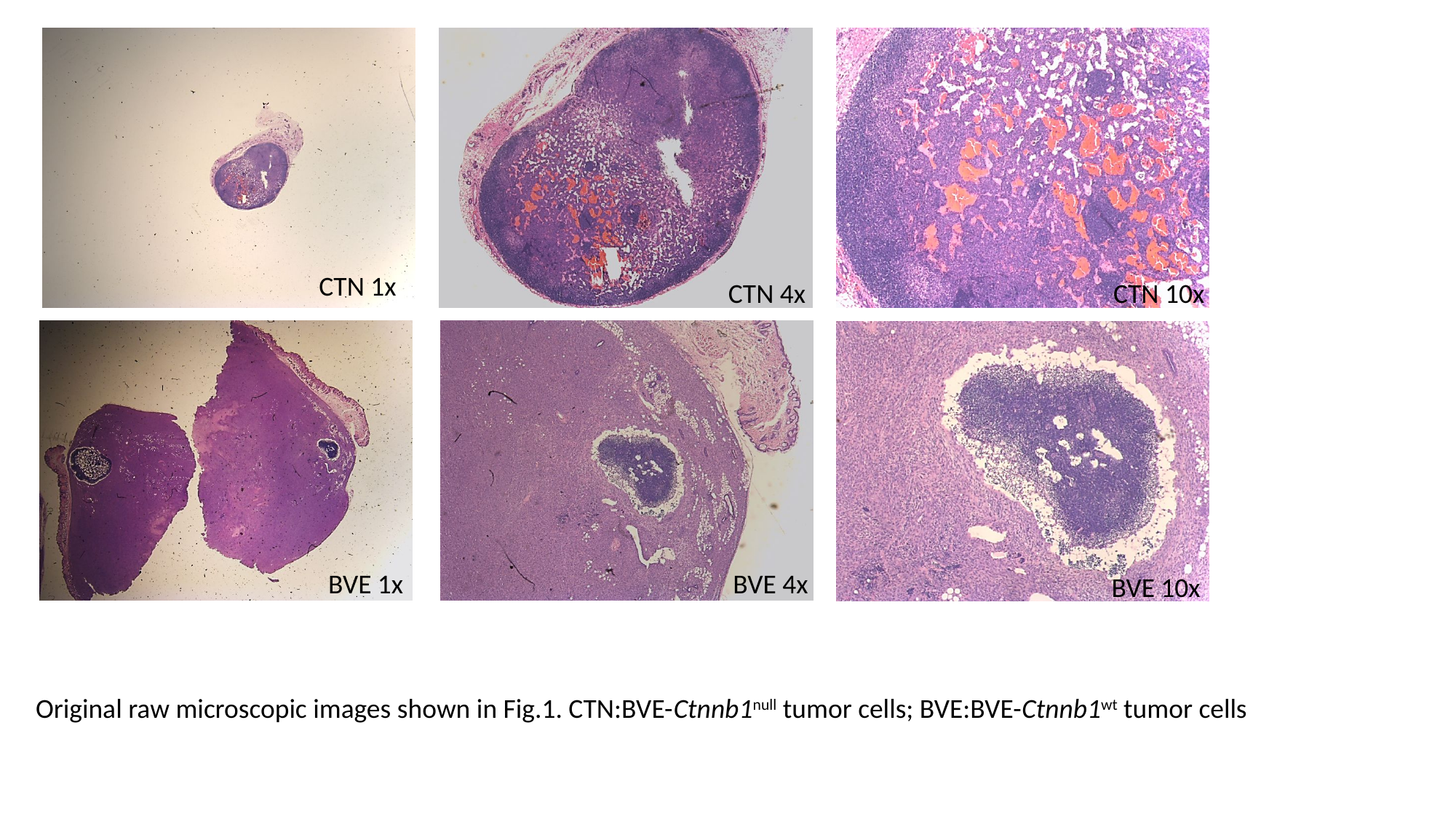

CTN 1x
CTN 4x
CTN 10x
BVE 1x
BVE 4x
BVE 10x
Original raw microscopic images shown in Fig.1. CTN:BVE-Ctnnb1null tumor cells; BVE:BVE-Ctnnb1wt tumor cells
